# Metabolomic analysis of porcine intestinal epithelial cells during swine acute diarrhea syndrome coronavirus infection

**DOI:** 10.3389/fcimb.2022.1079297

**Published:** 2022-12-01

**Authors:** Siying Zeng, Ouyang Peng, Fangyu Hu, Yu Xia, Rui Geng, Yan Zhao, Yihong He, Qiuping Xu, Chunyi Xue, Yongchang Cao, Hao Zhang

**Affiliations:** ^1^ State Key Laboratory of Biocontrol, Life Sciences School, Sun Yat‐sen University, Guangzhou, China; ^2^ Guangdong Provincial Key Laboratory of Malignant Tumor Epigenetics and Gene Regulation, Sun Yat‐sen University, Guangzhou, China

**Keywords:** SADS-CoV, metabolomics, ferroptosis, IPI-FX, TCA cycle

## Abstract

Swine acute diarrhea syndrome coronavirus (SADS-CoV) is an enveloped, positive single-stranded RNA virus belonging to *Coronaviridae* family*, Orthocoronavirinae* subfamily*, Alphacoronavirus* genus. As one of the main causes of swine diarrhea, SADS-CoV has brought huge losses to the pig industry. Although we have a basic understanding of SADS-CoV, the research on the pathogenicity and interactions between host and virus are still limited, especially the metabolic changes induced by SADS-CoV infection. Here, we utilized a combination of untargeted metabolomics and lipomics to analyze the metabolic alteration in SADS-CoV infected cells. Significant changes were observed in 1257 of 2225 metabolites identified in untargeted metabolomics, while the number of lipomics was 435 out of 868. Metabolic pathway enrichment analysis showed that amino acid metabolism, tricarboxylic acid (TCA) cycle and ferroptosis were disrupted during viral infection, suggesting that these metabolic pathways may partake in pathological processes related to SADS-CoV pathogenesis. Collectively, our findings gain insights into the cellular metabolic disorder during SADS-CoV infection, offer a valuable resource for further exploration of the relationship between virus and host metabolic activities, and provide potential targets for the development of antiviral drugs.

## Introduction

Coronaviruses (CoVs) are enveloped, positive single-stranded RNA viruses belonging to *Orthocoronaviridae* subfamily, *Coronaviridae* family, *Nidovirales* order (Cui et al., 2019). Over the past two decades, CoVs have caused three severe epidemics in human population, including severe acute respiratory syndrome coronavirus (SARS-CoV) (Drosten et al., 2003), Middle east respiratory syndrome coronavirus (MERS-CoV) (Zaki et al., 2012) and SARS-CoV-2 (Zhou et al., 2020a), posing a public health threat to human. Of note, several CoVs outbreaks have also occurred in pig herds in recent years, such as porcine epidemic diarrhea virus (PEDV) in 2010, porcine deltacoronavirus (PDCoV) in 2014 and swine acute diarrhoea syndrome coronavirus (SADS-CoV) in 2017 (Zhou et al., 2019). A growing number of evidence supports that CoVs have a propensity for crossing species barriers, for instance, the ongoing SARS-CoV-2 is believed to be of bat origin (Zhou et al., 2020a). Given the high density of animal populations in modern farms and close contact with humans, a broad general understanding of animal CoVs is essential to prevent future zoonosis events and protect livestock and human health.

SADS-CoV, also known as porcine enteric alphacoronavirus (PEAV) and swine enteric alphacoronavirus (SeACoV), is a member of *Alphacoronavirus* (Gong et al., 2017; Pan et al., 2017; Zhou et al., 2018). Genomic sequence analysis demonstrated that SADS-CoV shared more than 90% sequence similarity with bat HKU2-CoV, suggesting that SADS-CoV was transmitted to piglets from bats across species (Zhou et al., 2018; Yang et al., 2020). Moreover, it was reported that SADS-CoV possessed a broad species tropism *in vitro* and is able to infect diverse species cell lines, including pigs, bats, rodents, chickens and humans (Hu et al., 2015; Yang et al., 2019; Edwards et al., 2020; Luo et al., 2021). Furthermore, *in vivo* evidence confirmed that in addition to pigs, mice and chickens were also susceptible to SADS-CoV infection (Mei et al., 2022). These highlight an urgent need for a better understanding of the mechanisms of viral infection. SADS-CoV is a newly discovered enteric coronavirus that causes severe diarrhea and vomiting, with a mortality rate of nearly 90% in piglets under 5 days old (Gong et al., 2017; Pan et al., 2017; Zhou et al., 2018). The current research showed that infection with SADS-CoV induced apoptosis in ileal epithelial cells *in vivo* and Vero and IPI-2I cells *in vitro* (Zhang et al., 2020). To combat host immune defenses, viruses have evolved a series of strategies to modulate host signals in favor of their own survival and reproduction. The interaction between SADS-CoV N protein and RIG-I led to the proteasome dependent degradation of RIG-I, which subsequently inhibited the host interferon (IFN) response (Zhou et al., 2020b). Another research revealed that SADS-CoV N protein counteracted the interaction between TNF receptor-associated factor 3 (TRAF3) and TANK binding kinase 1 (TBK1), thereby reducing TBK1 activation and IFN-β production (Zhou et al., 2021). However, research on SADS-CoV remains limited and no effective antivirals or vaccines are available for the treatment of viral infection.

As is known to all, viruses are parasitic organisms without their own metabolism, and the viral propagation depends on the cell energy and metabolic resources of the host. Mice injected with respiratory syncytial virus (RSV) showed a decrease in plasma triacylglycerol (TAGs) and free fatty acids (FFAs), and an increase in plasmalogen phosphatidylcholines (PCps) and phosphatidylethanolamines (PEps) (Shan et al., 2018). Dysregulation of amino acid metabolism had been observed in patients infected with SARS-CoV-2, in particular the metabolites of the urea cycle (Li et al., 2021; Jia et al., 2022). Plasma lipidome revealed that exosomes were increasingly abundant in monosialodihexosyl ganglioside (GM3) with disease severity in COVID-19 (Song et al., 2020). The abnormal metabolic disorder reflects the propensity of the virus to the metabolic environment. By altering cellular metabolic activity, viruses can obtain the substrates and ATP needed for rapid replication and assembly (Bley et al., 2020). Influenza A virus (H1N1) enhanced glycolysis during infection, while pharmacological inhibition of the glycolytic pathway significantly reduced its replication, indicating that glycolysis was required for H1N1 infection (Ren et al., 2021). Dengue virus (DENV) infection induced autophagy dependent processing of triglycerides and lipid droplets to release free fatty acids, which were necessary for effective replication of DENV (Heaton and Randall, 2010). Thus, metabolomics approaches are powerful tools for gaining insight into aberrant regulation of host physiological processes during infection, which provide clues for further study of pathogenic mechanisms.

Metabolomics is a rapidly developing field that combines advanced analytical chemistry with complex statistical methods to comprehensively characterize metabolomes (German et al., 2005). Mass spectrometry (MS) is commonly used for metabolomic analysis because of its excellent sensitivity and ability to detect and quantify a large variety of molecules from cells, tissues, or whole organisms (Aksenov et al., 2017; Misra, 2020). Technological evolution in metabolomics have led to a renewed interest in the role of metabolism and small molecule metabolites in many biological processes. It is generally accepted that the metabolome is the closest representative of the phenotype, which provides insights into the cellular response to some stimuli, such as starvation, hypoxia, and infection (Bauermeister et al., 2022). Therefore, metabolomics can be applied to identify predictive biomarkers and is increasingly used as a blueprint for understanding the pathophysiological mechanisms of various diseases.

Herein, porcine ileum epithelial cell line, IPI-FX cells were collected and subjected to untargeted metabolome and lipidome to analyze metabolic changes after SADS-CoV infection. We provide a systematic view of the metabolic profiles in infected cells, which uncover host metabolic response to SADS-CoV. Dysregulation of numerous intracellular metabolites was observed following viral infection. Integrative analysis revealed that these altered metabolites mainly partook amino acid metabolism, tricarboxylic acid (TCA) cycle and ferroptosis pathway. This work may provide valuable information for the subsequent development of therapeutic strategies for metabolic disorders caused by viruses.

## Results

### SADS-CoV infection in IPI-FX cells and study design

**Figure 1:**
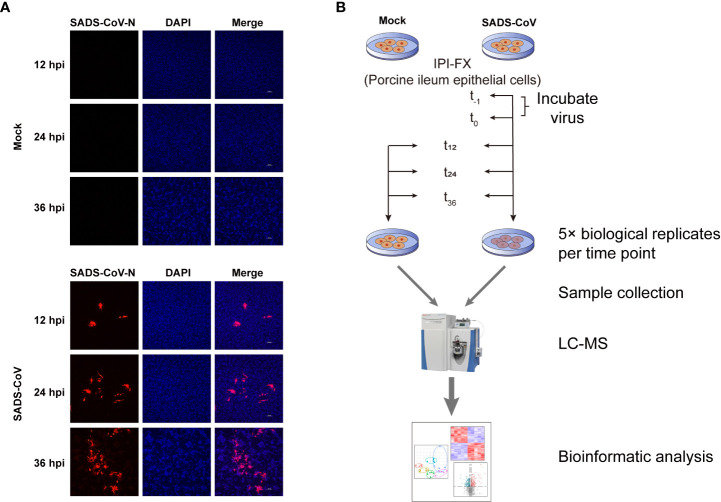
Workflow and detection of SADS-CoV replication in IPI-FX cells. **(A)** Immunofluorescence staining of SADS-CoV-infected or mock-infected IPI-FX cells at 12, 24, or 36 hpi. Viral N protein (red) was detected through indirect immunofluorescence assay. Nuclei (blue) was shown by 4′,6-diamidino-2-phenylindole (DAPI) staining. **(B)** Schematic diagram of study design of untargeted metabolomics and lipomics.

To investigate the metabolites profiles during SADS-CoV infection, we first confirmed SADS-CoV replication in porcine ileum epithelial cell line, IPI-FX, at a MOI of 0.1 for a period of 12, 24, and 36 hours (**Figure 1A**). Mock-infected cells were used as controls. The results of indirect immunofluorescence assay (IFA) showed that the virus could be detected at 12 hours post-infection (hpi), and the red fluorescence increased with time, indicating that SADS-CoV had successfully proliferated in the IPI-FX cells. An untargeted metabolomics and lipomics approach based on liquid chromatography-mass spectrometry (LC-MS) were used to identify the metabolic characteristics related to SADS-CoV infection (**Figure 1B**). Infected and mock-infected cells were harvested at 12, 24 and 36 hours, with 5 biological replicates at each time point. Data were collected for subsequent research.

### Overview of untargeted metabolomics and lipomics

**Figure 2:**
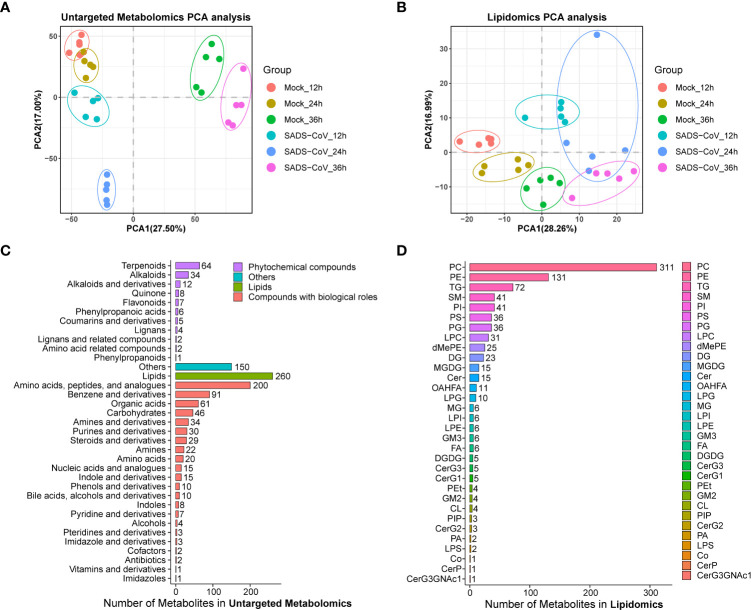
Overview of the untargeted metabolomics and lipomics. Principal component analysis (PCA) plot of untargeted metabolomics **(A)** and lipomics **(B)** in SADS-CoV infected and mock-infected cells. Each dot represents an individual sample. Metabolite classification of untargeted metabolomics **(C)** and lipomics **(D)**.

Principal component analysis (PCA) for quality control (QC) samples can be used to investigate the overall distribution of samples in each group and the stability of the whole analysis process. In general, the data revealed consistency and repeatability for both untargeted metabolomics and lipomics, and there was a noticeable distinction between the infected cells and those of controls (**Figures 2A, B**). It is noteworthy that the distinctions at 12 hpi and 24 hpi were more obvious than at 36 hpi, which might indicate that intracellular metabolite differences reduced in the late stage of infection. In total, we obtained 11,375 metabolites from the data of the untargeted metabolomics, but only 2,225 had identification information. The lipidome had much less data, only 868 metabolites but all of them were identified. By classifying the metabolites, it was found that most of them in non-targeted metabolomics belonged to lipids and amino acid, peptide, and analogues, while most of the molecules in lipidome were phosphatidylcholine (PC) and phosphatidylethanolamine (PE) (**Figures 2C, D**). Overall, we have successfully obtained a large amount of available information from metabolomics.

### Trend analysis of metabolites

**Figure 3:**
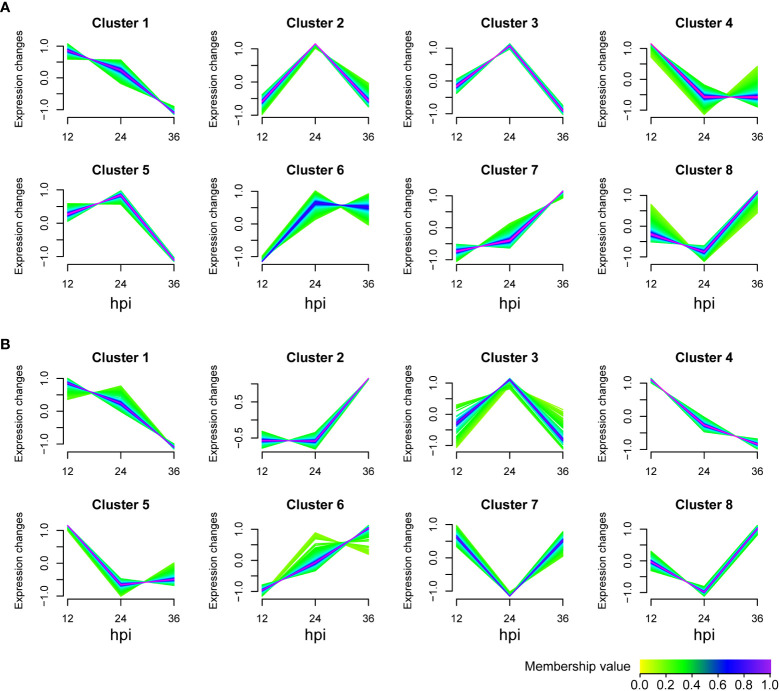
Metabolite expression trend analysis. Graphs of clusters in SADS-CoV-infected IPI-FX cells. **(A)** Untargeted metabolomics. **(B)** Lipomics. Metabolites in the same cluster have similar expression patterns. Green or cyan colored lines correspond to genes with low membership value; blue or purple colored lines correspond to genes with high membership value.

To determine how IPI-FX cells respond to SADS-CoV infection, the identified metabolites in viral infection groups were classified into 8 clusters using the Mfuzz package (v 2.48.0) according to the expression information with the time (**Figure 3**). The metabolites of untargeted metabolomics classified in clusters 1, 2, 3, 4, and 5 were downregulated, whereas the metabolites in clusters 6, 7, and 8 were upregulation (**Figure 3A**). For lipomics, clusters 1, 3, 4, and 5 showed a downregulation trend, while clusters 2, 6, 7 and 8 showed upregulated expression (**Figure 3B**). Those metabolites clustered in one group are more likely to be considered to belong to the same functional set.

### Significant changes of metabolites were observed following SADS-CoV infection in IPI-FX cells

**Figure 4:**
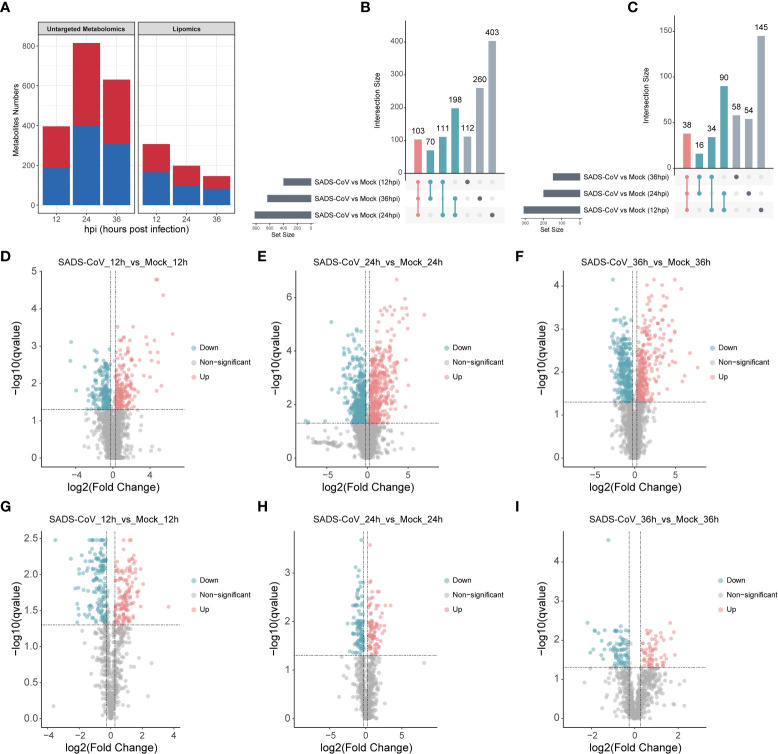
Multivariate analysis of metabolic profiles induced by SADS-CoV infections. **(A)** Number of differential metabolites in untargeted metabolomics (left) and lipomics (right). The overlap among the altered metabolites in untargeted metabolomics **(B)** and lipomics **(C)** were shown in Venn diagram. Volcano plots of untargeted metabolomics **(D–F)** and lipomics **(G–I)** for the infected and mock-infected cells at the indicated time points. Each point represents a metabolite. Red, upregulation; blue, downregulation; gray, non-significant.

Comparing the metabolomic data between SADS-CoV infected IPI-FX cells and controls, there were significant differences in the level of 1257 metabolites in untargeted metabolomics, while in the lipomics, there were 435 significantly changed metabolites (Fold-Change ≥ 1.2 or ≤ 0.83, q-value < 0.05) (**Figure 4A**). The number of upregulated and downregulated metabolites at each time point was about equal. In order to visually compare common and unique differential metabolites between different comparison groups, Venn diagrams were drawn for all comparison groups with different metabolites (**Figures 4B, C**). The results showed that the metabolites were altered in abundance in different time points compared to those in uninfected cells. Volcano plots were also used to visualize differential metabolites (**Figures 4D–I**). Each point in the volcanic map represents a metabolite. As a whole, we demonstrate that a large number of intracellular metabolites are significantly altered during viral infection.

### Analysis of metabolic pathways in untargeted metabolomics

**Figure 5:**
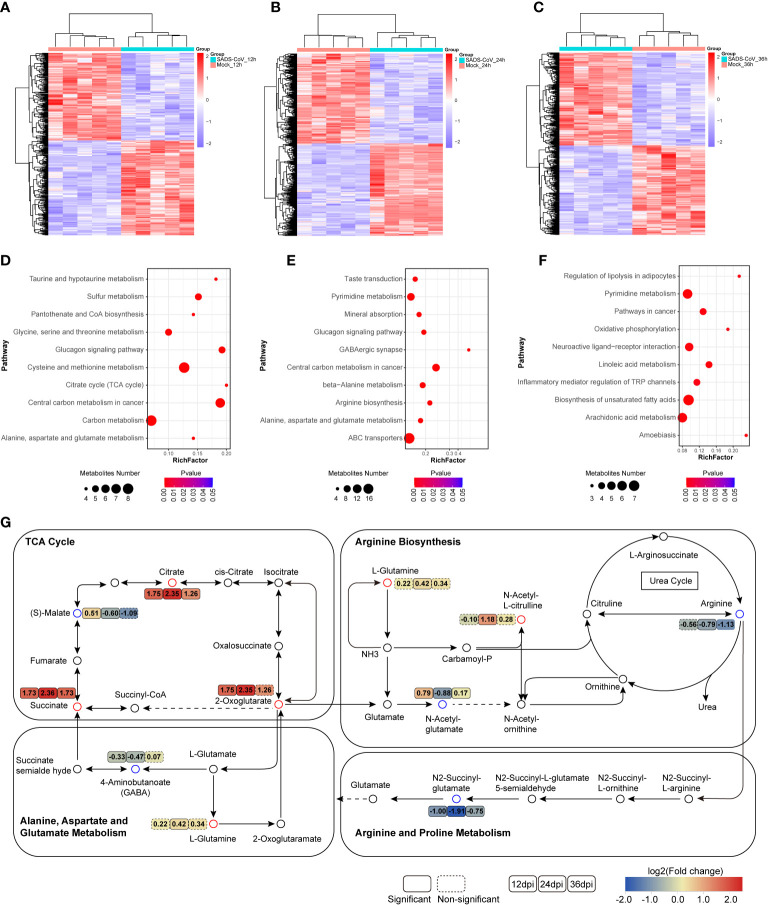
Significant changes in metabolites and metabolic pathways in untargeted metabolomics. **(A–C)** Heatmap of hierarchical clustering analysis of differential metabolites at different time points. **(D–F)** Bubble plots of metabolic pathway enrichment analysis for SADS-CoV infected cells at 12 hpi **(D)**, 24 hpi **(E)** and 36 hpi **(F)**. The dot size represents the numbers of differential metabolites annotated in this pathway. **(G)** Schematic overview of metabolic pathways among amino acid metabolism, the urea cycle and TCA cycle. Red, upregulation; blue, downregulation.

Moreover, to elucidate the intracellular metabolic characteristics after SADS-CoV infection in IPI-FX cells, we further analyzed the data of the untargeted metabolome. Heatmap analysis depicting hierarchical clustering of the dysregulated metabolite data from a comparison group intuitively showed that the metabolites in the two groups of samples had different dynamic expression profiles (**Figures 5A–C**). To uncover the metabolic pathways potentially involved in the process of SADS-CoV infection, pathway enrichment analysis of differential metabolites based on the Kyoto encyclopedia of genes and genomes (KEGG) database was employed (**Figures 5D–F**). The top 10 metabolic pathways with the smallest p value were drawn as bubble charts. On the whole, we found that a striking feature of SADS-CoV infection was a significant change in amino acid metabolism, including glycine, serine and threonine metabolism, cysteine and methionine metabolism, alanine, aspartate and glutamate metabolism, and arginine biosynthesis.

The urea cycle is responsible for amino acid metabolism, especially closely related to arginine and glutamate metabolism (**Figure 5G**). Metabolomic data showed that arginine levels were downregulated and glutamine levels were upregulated in SADS-CoV infected cells, suggesting that these metabolites might have a certain function in the process of virus replication. Notably, amino acid metabolism is always related to energy metabolism. The citrate cycle (TCA cycle), an important energy metabolism, was active at 12 hpi (**Figures 5D, G**). We hypothesized that the virus might disrupt the TCA cycle to rapidly obtain the ATP needed for its own replication during the early stage of infection. In addition, obvious changes in lipid response and inflammatory pathways were observed at 36 hpi, which may be associated with the aggravation of cytopathological characteristics in the late stage of infection (**Figure 5F**). Taken together, SADS-CoV infection led to disorder of a large number of cellular metabolic pathways, especially amino acid metabolism.

### Analysis of metabolic pathways in lipomics

**Figure 6:**
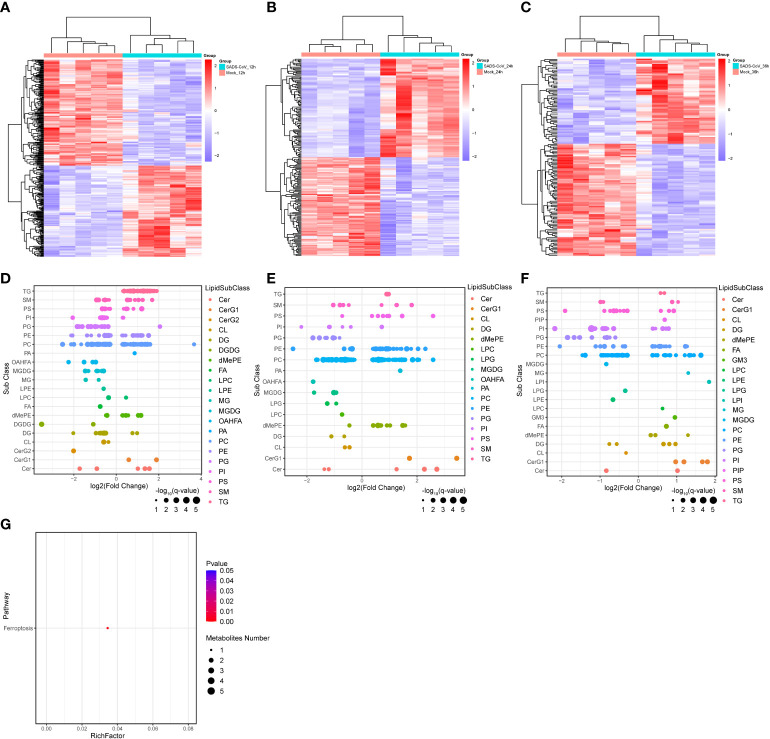
Significant changes in metabolites and metabolic pathways in lipomics. **(A–C)** Heatmap of hierarchical clustering analysis of differential metabolites at different time points. **(D–F)** Bubble charts were used to display the expression patterns of differential metabolites at 12 hpi **(D)**, 24 hpi **(E)** and 36 hpi **(F)**. Each point in the figure represents a different lipid. **(G)** Metabolic pathway enrichment analysis for SADS-CoV infected cells at 12 hpi.

Similarly, we further analyzed the lipomics data to explore important lipid metabolites or metabolic pathways during SADS-CoV infection. Cluster analysis of differential metabolite expression level also demonstrated that the metabolites between infected and mock-infected cells had different dynamic expression profiles, speculating that the virus might organize its own metabolic networks to serve self-replication (**Figures 6A–C**). The expression patterns of differential metabolites at different time points were analyzed by Bubble chart (**Figures 6D–F**). In particular, we found that triglyceride (TG) and monoglycosylceramide (CerG1) showed an up-regulated trend at all three time points of infection. The former belongs to glycerolipids, while the latter is a kind of saccharolipids. This observation suggested that these two metabolites might be involved in the process of viral replication. Furthermore, to investigate the differential metabolic pathway related to SADS-CoV infection, we also conducted metabolic pathway enrichment analysis in lipomics (**Figure 6G**). Unexpectedly, only ferroptosis was observed to be enriched at 12 hpi. We suspect that this may be because the functions of many lipid molecules have not been clearly studied and there is less annotation information available for analysis.

### SADS-CoV inhibited ferroptosis in IPI-FX cells in the early stage of infection

**Figure 7:**
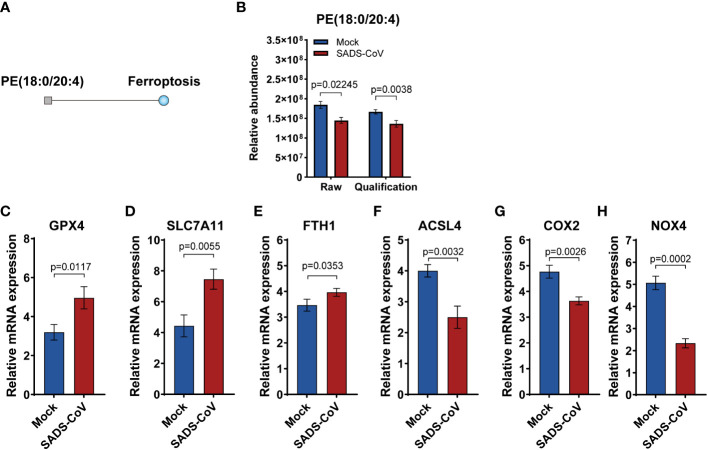
Ferroptosis was suppressed in the early stage of SADS-CoV infection. **(A)** Metabolic pathway enrichment analysis network plot of ferroptosis at 12 hpi. Square indicates differential metabolite; blue indicates downregulation. **(B)** Relative abundance of PE (18:0/20:4) in the discovery and validation samples at 12 hpi. **(C–H)** Some markers of ferroptosis were detected in SADS-CoV infected cells at 12 hpi. **(C)** GPX4; **(D)** SLC7A11; **(E)** FYH1;**(F)** ACSL4; **(G)** COX2; **(H)** NOX4.

Ferroptosis is a novel pattern of cell death characterized by excessive accumulation of iron and overwhelming lipid peroxidation (Tang et al., 2021). In order to study the specific details of ferroptosis in lipomics, we performed metabolic pathway enrichment analysis network plot. The results revealed that in the early stage of SADS-CoV infection, ferroptosis was suppressed and the expression of phosphatidylethanolamine (PE) (18:0/20:4) was downregulated (**Figures 7A, B**). Besides, we detected the expression levels of some markers of ferroptosis in SADS-CoV infected cells, and found that positive regulators expression (Acyl-CoA synthetase long chain family member 4, ACSL4; Cytochrome c oxidase subunit II, COX2; NADPH oxidase 4, NOX4) were down-regulated, while negative regulators expression (Glutathione peroxidase 4, GPX4; Solute carrier family 7 member 11, SLC7A11; Ferritin heavy chain 1, FYH1) were up-regulated (**Figures 7C–H**). Taken together, we preliminarily demonstrated that the intracellular ferroptosis was inhibited during the early stage of SADS-CoV infection in IPI-FX cells.

## Discussion

The development of metabolomics provides a powerful means for rapid and comprehensive understanding of metabolic changes during viral infection. In this study, we systematically demonstrated the dynamic profiles of metabolic alterations in SADS-CoV infected cells through untargeted metabolomics and lipomics. The datasets showed that there were 2,225 and 868 identified metabolic molecules in untargeted metabolomics and lipomics, of which 1257 in the former and 435 in the latter were differentially changed. The large number of dysregulated metabolites indicated metabolic reprogramming of host cells during virus infection. Using pathway enrichment analysis, we found that a lot of metabolic pathways, including amino acid metabolism, TCA cycle, pyrimidine and purine metabolism, lipolysis and ferroptosis were changed significantly in the infected cells, suggesting potential roles of these pathways in virus replication. A previous study conducted untargeted metabolomic analysis in the small intestine of infected piglets and uncovered bile acid (BA) as an important factor in promoting SADS-CoV replication (Yang et al., 2022). Another research revealed that African swine fever virus (AFSV) could modulate lactate production to facilitate its own replication, and inhibitor of lactate dehydrogenase resulted in reduced proliferation of ASFV (Xue et al., 2022). Studying the mechanisms by which viruses regulate host cell metabolism and designing new drugs targeting altered metabolic pathways have become an important preventive or therapeutic strategy.

A prominent feature of the metabolic disorder in SADS-CoV infected cells was the dysregulation of amino acid metabolism. Amino acid metabolism has a profound impact on cellular functions, not only on protein synthesis, but also on many other processes driving growth and proliferation (Kelly and Pearce, 2020). A growing number of reports have demonstrated a strong link between viral infection and amino acid metabolism. It was found that glutathione and its related metabolites, as well as some amino acids, such as aspartate and glutamate, altered significantly with the infectious dose of Enterovirus 71, while knockdown of glutaminase and glutamate dehydrogenase expression drastically reduced the viral replication (Cheng et al., 2020). The urea cycle was dysregulated with increased ornithine and decreased arginine in the serum of COVID-19 patients, indicating that metabolic reprogramming might be involved in the progress of COVID-19 disease (Jia et al., 2022). Moreover, supplementation of arginine modulated the inflammatory cytokine release by isolated peripheral blood mononuclear cells (PBMCs) derived from SARS-CoV-2-infected rhesus macaques (Xiao et al., 2021), suggesting that arginine was a potential target for developing therapeutic drugs.

Likewise, there exist specific changes in glutathione and arginine metabolism in our study. Compared with the control group, glutathione and glutamine level were significantly elevated after SADS-CoV infection. The dysregulated metabolites indicate that they may play a certain role in the virus life cycle and may be limiting factors for virus replication. In terms of arginine, a decreased level of this metabolite was observed during SADS-CoV infection in the present study. Interestingly, some other CoVs, including SARS-CoV-2, transmissible gastroenteritis virus (TGEV) and infectious bronchitis virus (IBV), also interfere with arginine metabolism, indicating that arginine may have similar functions in coronaviruses life cycles (Lee et al., 2002; Xia et al., 2018; Jia et al., 2022). In addition to participating in the urea cycle, arginine is also the substrate of various nitric oxide synthase (NOS), which converts it into citrulline and nitric oxide (NO) (Grimes et al., 2021). Given that NO is a critical mediator of the innate inflammatory immune response induced by virus infections (Ricciardolo et al., 2006), it is worth exploring whether SADS-CoV can modulate NO production by regulating arginine level so as to create a beneficial environment for self-replication.

Furthermore, we found that the ferroptosis pathway, an iron-dependent cell death characterized by excessive accumulation of lipid reactive oxygen species (ROS) and lipid peroxidation (Wu et al., 2021), was differentially changed in infected cells in lipomics. In recent years, ferroptosis has rapidly attracted attention because of its unique death pattern and potential value in clinical applications. However, researches on the relationship between viruses and ferroptosis are still limited. It was reported that human enterovirus and SARS-CoV-2 could induce ferroptosis and the inhibition of this pathway decreased the viral load (Kung et al., 2022). In our study, analysis of lipomics data and subsequent validation showed that the pro-ferroptosis PE (18:0/20:4) was downregulated, suggesting that SADS-CoV inhibited ferroptosis in IPI-FX cells in the early stage of infection. PE is a non-bilayer forming phospholipid that can be oxidized by lipoxygenase (LOX) to generates doubly and triply-oxygenated (15-hydroperoxy)-diacylated PE specie, which acts as a death signal (Kagan et al., 2017). Cell death is thought to be a host defense strategy against pathogen infection (Gradi et al., 1998). Thus, we speculate that SADS-CoV may inhibit ferroptosis at an early stage of infection to create an environment conducive to proliferation and survival. It is noteworthy that SADS-CoV induced significant apoptosis in the late stage of infection (Zhang et al., 2020). Therefore, it would be interesting to explore the link between different cell deaths regulated by SADS-CoV.

In conclusion, our metabolomic data presented here displayed a comprehensive untargeted metabolomics and lipomics analysis of mock-infected and SADS-CoV infected IPI-FX cells. SADS-CoV infection led to abnormal expression levels of a great quantity of metabolites and reprogramming of metabolic pathways. In particular, we found significant changes in amino acid metabolism, TCA cycle and ferroptosis, indicating that they may play important roles during viral infection. What is insufficient is that our study was limited to the cellular level and could not fully reflect the real situation of virus infection in animals. And we have only made preliminary verification for metabolites or metabolic pathways with significant differences, without further in-depth exploration. Nevertheless, our work still provides clues for further research on the mechanism of SADS-CoV infection, and guides the design of new therapeutic strategies targeting metabolic pathways.

## Materials and methods

### Cells and viruses

IPI-FX (porcine intestinal epithelial cells) were kindly provided by Prof. Shaobo Xiao (Huazhong Agricultural University) (Wang et al., 2019), which were grown in Dulbecco’s modified Eagle’s medium (DMEM, Gibco, New York NY, USA) containing 10% fetal bovine serum (FBS, Gibco, New York NY, USA), 100 U/mL penicillin, and 100 mg/mL streptomycin. SADS-CoV strain GDS04 (NCBI accession number: MF167434) was isolated and propagated in our laboratory.

### Immunofluorescence microscopy

IPI-FX cells were infected with the SADS-CoV at an MOI of 0.1 for 12, 24, or 36 hours. After collection, cells were fixed with pre-cooling methanol for 15 min and then washed with phosphate-buffered saline (PBS). Cells were then incubated with Triton X-100 (0.5%) for 15 min and following PBS washing. After blocked with 1% bovine serum albumin (BSA, Sangon, Shanghai, China) for 1 hour, cells were incubated with primary antibodies against SADS-CoV N protein (prepared and preserved in our laboratory) for 1 hour at room temperature. After PBS washing, cells were incubated with a 1:500 dilution of Cy3-conjugated goat anti-mouse IgG secondary antibodies (Abcam, Boston MA, USA) for 1 hour, following by 4’,6-diamidino-2-phenylindole dihydrochloride (DAPI, Invitrogen, Carlsbad CA, USA) staining for another 5 min. The cells were finally washed with PBS and observed using a fluorescence microscope (Nikon, Tokyo, Japan).

### Chemicals and reagents

LC-MS-level methanol, acetonitrile and formic acid were purchased from Thermo Fisher Scientific (Waltham MA, USA); ammonia formate was purchased from Honeywell Fluka (Sandy UT, USA). Ultrapure water was obtained from a Millipore system (Billerica MA, USA).

d_3_-Leucine, ^13^C_9_-Phenylalanine, d_5_-Tryptophan, ^13^C_3_-Progesterone were used as internal standard (#1) for untargeted metabolomics. MG 18:1(d7), 2 μg/mL; PS 15:0-18:1(d7), PE 15:0-18:1(d7), LPE 18:1(d7), 5 μg/mL each sample; PA 15:0-18:1(d7), 7 μg/mL; PI 15:0-18:1(d7), DG 15:0-18:1(d7), 10μg/mL each sample; LPC 18:1(d7), 25 μg/mL; PG 15:0-18:1(d7), SM d18:1-18:1(d9), 30 μg/mL each sample; TG 15:0-18:1(d7)-15:0, 55 μg/mL; cholesterol(d7), 100 μg/mL; PC 15:0-18:1(d7), 160 μg/mL; CE 18:1(d7), 350 μg/mL were used as internal standard (#2) for lipomics.

### Metabolite extraction for untargeted metabolomics

25 mg samples were weighed and added into 2 mL thickened centrifuge tubes, simultaneously, 2 magnetic beads were added as well. Thereafter, 10 μL of the prepared internal standard (#1) was added to each sample at the same time. Then 800 µL of precooled extraction reagent (methanol: acetonitrile: water (2:2:1, v/v/v)) was added, samples were grinded with TissueLyser at 50 Hz for 5 min, then were placed at -20°C for 2 hours. Samples were centrifuged at 25,000 × g for 15 min at 4°C and 600 µL of each sample were added in split-new EP tubes and frozen dry. 120 μL of 50% methanol were added to the dried samples and mixture was shaken until completely dissolved. And then samples were centrifuged at 25,000 × g for 15 min at 4°C and 600 µL of each sample were added in split-new EP tubes. 10 μL of each sample was taken and mixed as QC samples.

### Metabolite extraction for lipomics

25 mg samples were weighed and added into 2 mL thickened centrifuge tubes, simultaneously, 2 magnetic beads were added as well. Two steel balls and 800 µL of pre-chilled dichloromethane/methanol (3:1, v/v) precipitant were added into each sample, as well as 10 µL of the prepared internal standard (#2). Then samples were implemented ice bath ultrasound for 10 minutes and refrigerated overnight at -20°C. Samples were centrifuged at 25,000 × g for 15 minutes at 4°C and 600 µL supernatant 600 was taken and drained in freezer dryer. 120µL lipid reconstituted solution (isopropanol: acetonitrile: water = 2:1:1) was reconstituted and shaken for 10 min. Then samples were implemented ice bath ultrasound for 10 minutes and centrifuged at 25,000 × g for 15 minutes at 4°C. 10 μL of each sample was taken and mixed as QC samples.

### UPLC-MS analysis for untargeted metabolomics

Waters UPLC I-Class Plus (Waters, Milford MA, USA) and tandom Q Exactive high resolution mass spectrometer (Thermo Fisher Scientific, Waltham MA, USA) was employed for separation and detection of metabolites.

Chromatographic separation was performed on an ACQUITY UPLC BEH C18 column (Waters, USA) and the column temperature was maintained at 45°C. In the positive mode, the mobile phase consisted of 0.1% formic acid (A) and acetonitrile (B). The mobile phase consisted of 10 mM ammonium formate (A) and acetonitrile (B) in the negative mode. The gradient conditions were as follows: 0-1 min, 2% B; 1-9 min, 2-98% B; 9-12 min, 98% B; 12-12.1 min, 98% B to 2% B; and 12.1-15 min, 2% B. The flow rate was 0.35 mL/min and the injection volume was 5 μL.

Q Exactive perform was used for primary and secondary mass spectrometry data acquisition. The full scan range was 70-1050 m/z with a resolution of 70,000, and the automatic gain control (AGC) target for MS acquisitions was set to 3e6 with a maximum ion injection time of 100 ms. Top 3 precursors were selected for subsequent MSMS fragmentation with a maximum ion injection time of 50 ms and resolution of 17,500, the AGC was 1e5. The stepped normalized collision energy was set to 20, 40 and 60 eV. ESI parameters were set as follows: Sheath gas flow rate: 40; Aux gas flow rate: 10; positive-ion mode Spray voltage(|KV|): 3.80; negative-ion mode Spray voltage(|KV|): 3.20; Capillary temperature: 320°C; Aux gas heater temperature: 350°C.

### UPLC-MS analysis for lipomics

Equipments for separation and detection of metabolites were the same as that for untargeted metabolomics above.

Chromatographic separation was performed on CSH C18 column (Waters, USA). At positive ion mode with mobile phase A consisting 60% acetonitrile in water + 10 mM ammonium formate + 0.1% formic acid and mobile phase B consisting 90% isopropanol + 10% acetonitrile + 10 mM ammonium formate + 0.1% formic acid. At positive ion mode, with mobile phase A consisting 60% acetonitrile in water + 10 mM ammonium formate and mobile phase B consisting 90% isopropanol + 10% acetonitrile + 10 mM ammonium formate. The column temperature was maintained at 55°C. The gradient conditions were as follows: 40-43% B over 0~2 min, 43-50% B over 2-2.1 min, 50-54% B over 2.1-7 min, 54-70% B over 7-7.1 min, 70-99% B over 7.1-13 min, 99-40% B over 13-13.1 min, held constant at 99-40% B over 13.1~15 min and washed with 40% B over 13.1-15 min. The flow rate was 0.4 mL/min and the injection volume was 5 μL.

Q Exactive perform was used for primary and secondary mass spectrometry data acquisition. The stepped normalized collision energy was set to 15, 30 and 45 eV. All other parameters were set the same as that above.

### Metabolite ion peak extraction and metabolite identification for untargeted metabolomics

After importing the off-line data of mass spectrometry into compound discoverer 3.2 (Thermo Fisher Scientific, USA, https://mycompounddiscoverer.com/) software and analyzing the mass spectrometry data in combination with bmdb (BGI metabolome database), mzcloud database and chemspider online database, a data matrix containing information such as metabolite peak area and identification results will be obtained. Paramenters were set as: parent ion mass deviation: < 5 ppm; mass deviation of fragment ions: < 10 ppm; retention time deviation: < 0.2 min.

### Metabolite ion peak extraction and metabolite identification for lipomics

After importing the off-line data of mass spectrometry into lipidsearch (v 0.4.1) (Thermo Fisher Scientific, USA, https://www.thermofisher.cn/order/catalog/product/OPTON-30880) software for mass spectrometry data analysis, the identification results and results of lipid containing molecules will be obtained, the data matrix of quantitative results and other information, and then the table will be further analyzed and processed. Paramenters were set as: identification type: product; search quality deviation of parent ion and daughter ion: 5 ppm; response threshold: Relative response deviation of sub ions5.0%; peak lifting quality deviation: 5ppm; m-score: 5.0; c-score: 2.0; identification level selection: “A”, “B”, “C”, “D”; positive ion mode adduct form: [M+H]^+^, [M+NH4]^+^, [M+Na]^+^; negative ion mode adduct form: [M-H]^-^, [M-2H]^-^, [M+HCOO]^-^; retention time deviation: 0.1 min.

### Data preprocessing

Metabolites with more than 50% missing values in QC samples and more than 80% missing values in experimental samples were removed, and missing values were filled with k-Nearest Neighbor Algorithm (KNN). Data were normalized to obtain relative peak areas by Probabilistic Quotient Normalization, PQN (Di Guida et al., 2016). Quality control-based robust LOESS (Dunn et al., 2011) signal correction was used to correct Batch effect, then metabolites with a coefficient of variation larger than 30% on their Relative peak area in QC Samples were removed.

### Data visualization, enrichment and pathway analysis

Principal component analysis (PCA) was performed with R language (https://www.R-project.org/). R package Mfuzz (v 2.48.0) was used to cluster metabolite expression levels from samples at different time point (Kumar and Futschik, 2007). R package UpsetR (v 1.4.0) was employed to visualize intersecting sets (Lex et al., 2014). R package ggplot2 (v 3.3.6) was used to visualize the bar, bubble and volcano plots (Villanueva and Chen, 2019). For metabolic network mapping, the compound names were uploaded into the a web tool Chemical Translation Service (http://cts.fiehnlab.ucdavis.edu/batch) (Wohlgemuth et al., 2010) to obtain the PubChem Compound Identifiers (CID) and the Kyoto Encyclopedia of Genes and Genomes identifiers (KEGG ID). Metabolite enrichment analysis was performed with the web-based software MBROLE (v 2.0) (Lopez-Ibanez et al., 2016). Significantly changed metabolites matching the KEGG database were imputed, following overrepresentation analysis against the KEGG pathway module. Finally, annotations with q-values lower than 0.05 were considered significantly enriched.

### Quantitative real-time PCR

To analyze mRNA expression, 0.1 μg of total RNA was used to reverse transcribe in order to construct cDNA using the First Strand cDNA Synthesis Kit (TOYOBO, Tokyo, Japan) according the manufacturer’s protocol. Quantitative real-time PCR was performed using the SYBR Green Real-time PCR Master Mix (Yeasen, Shanghai, China) and a LightCycler480 II system (Roche, Basel, Switzerland). Amplification was performed as follows: 50°C for 2 min, 95°C for 10 min, followed by 40 cycles at 95°C for 15 s, 60°C for 15 s, and 72°C for 30 s. The relative expression values of mRNAs were normalized to that of GAPDH in each sample using the 2^−ΔΔCt^ method. Primers used for detecting ferroptosis markers were shown in **Table 1**.

**Table 1:** Primers used in this study.

Genes	Forward	Reverse
GPX4	5′-TGGCCTCTCAATGAGGCAAG-3′	5′-CCCTTGGGCTGGACTTTCAT-3′
SLC7A11	5′-TGCGGGTGACTACAATGGTC-3′	5′-GATCCCTGCTCCGATGATGG-3′
ACSL4	5′-TCTGTGCAGTAACTTGTGTGG-3′	5′-GCAGGGATACATCATCCCAGAT-3′
FTH1	5′-ATTTGCGCTGCACGTGGT-3′	5′-ATACTCGGCCATGCCATACTC-3′
COX2	5′-TACTGCACTCATGAGCCGTC-3′	5′-ATAAAGGCCAGGTCGTGTGG-3′
NOX4	5′-GGGAGATGGAGGGAAGCTTTA-3′	5′-GGCCCTCGGTTATACAGCAG-3′

## Data availability statement

The datasets presented in this study can be found in online repositories. The names of the repository/repositories and accession number(s) can be found in the article/**Supplementary Material**.

## Author contributions

Conceptualization, HZ and SZ. Methodology, SZ. Experiments, SZ, FH, OP, YX, YZ, RG and YH. Software, OP, and FH. Writing—original draft preparation, SZ and OP. Writing—review and editing, HZ and QX. Supervision, HZ, YC and CX. Project administration, HZ. Funding acquisition, HZ, YC and CX. All authors contributed to the article and approved the submitted version.

## Funding

This work was supported by the National Key Research and Development Program of China (No. 2021YFD1801102) and Guangdong Natural Science Foundation (No. 2018B030314003).

## Acknowledgments

We thank Professor Shaobo Xiao (Huazhong Agricultural University, Wuhan, China) for kindly providing us with porcine ileum epithelial cell line (IPI-FX).

## Conflict of interest

The authors declare that the research was conducted in the absence of any commercial or financial relationships that could be construed as a potential conflict of interest.

## Publisher’s note

All claims expressed in this article are solely those of the authors and do not necessarily represent those of their affiliated organizations, or those of the publisher, the editors and the reviewers. Any product that may be evaluated in this article, or claim that may be made by its manufacturer, is not guaranteed or endorsed by the publisher.
